# Overexpressed c-Myc Sensitizes Cells to TH1579, a Mitotic Arrest and Oxidative DNA Damage Inducer

**DOI:** 10.3390/biom12121777

**Published:** 2022-11-29

**Authors:** Sofia Henriksson, José Manuel Calderón-Montaño, Daniel Solvie, Ulrika Warpman Berglund, Thomas Helleday

**Affiliations:** 1 Science for Life Laboratory, Department of Oncology-Pathology, Karolinska Institute, 17165 Stockholm, Sweden; 2 Department of Pharmacology, Faculty of Pharmacy, University of Seville, 41012 Seville, Spain; 3 Theodor Boveri Institute, Department of Biochemistry and Molecular Biology, Biocenter, University of Würzburg, 97070 Würzburg, Germany; 4 Oxcia AB, Norrbackagatan 70C, 11334 Stockholm, Sweden

**Keywords:** MTH1, TH588, TH1579, c-Myc, replication stress, DNA damage, cell death, cancer

## Abstract

Previously, we reported that MTH1 inhibitors TH588 and TH1579 selectively induce oxidative damage and kill Ras-expressing or -transforming cancer cells, as compared to non-transforming immortalized or primary cells. While this explains the impressive anti-cancer properties of the compounds, the molecular mechanism remains elusive. Several oncogenes induce replication stress, resulting in under replicated DNA and replication continuing into mitosis, where TH588 and TH1579 treatment causes toxicity and incorporation of oxidative damage. Hence, we hypothesized that oncogene-induced replication stress explains the cancer selectivity. To test this, we overexpressed c-Myc in human epithelial kidney cells (HA1EB), resulting in increased proliferation, polyploidy and replication stress. TH588 and TH1579 selectively kill c-Myc overexpressing clones, enforcing the cancer cell selective killing of these compounds. Moreover, the toxicity of TH588 and TH1579 in c-Myc overexpressing cells is rescued by transcription, proteasome or CDK1 inhibitors, but not by nucleoside supplementation. We conclude that the molecular toxicological mechanisms of how TH588 and TH1579 kill c-Myc overexpressing cells have several components and involve MTH1-independent proteasomal degradation of c-Myc itself, c-Myc-driven transcription and CDK activation.

## 1. Introduction

In our lab, we generated mitotic MTH1 inhibitors TH588 and TH1579 and showed that these have anti-cancer properties, selectively killing transforming cancer cells and being well-tolerated in non-transformed cells [1,2]. Currently, TH1579 is being evaluated in several clinical trials (Eudnr 2016-00262480 and 2019-001221-27), and here we wanted to understand the molecular mechanism for the cancer selectivity of TH588 and TH1579. TH588 and TH1579 are mitotic MTH1 inhibitors and act via a dual mechanism, (i) causing mitotic arrest by disturbing microtubule polymerization [3] (likely both dependent on and independent of MTH1 [4]), which altogether increases ROS, and (ii) by inhibiting MTH1 in promoting the incorporation of 8-oxodGTP into DNA during mitotic replication [4,5]. In humans, the MTH1 enzyme is involved in protection against reactive oxygen species (ROS), where it hydrolyzes oxidized dNTPs, such as 8-oxodGTP into 8-oxodGMP, preventing the incorporation of this oxidized nucleotide into DNA [6]. Moreover, recent data have shown that the MTH1 protein binds tubulin and promotes microtubule polymerization and mitotic progression to avoid formation of oxidative DNA damage [4]. Different MTH1 inhibitors differentially affect microtubule polymerization. TH588 and TH1579 cause incorporation of oxidized dNTPs during mitosis in mitotic replication, an effect linked to MTH1 inhibition [5]. While ROS have been suggested to contribute to replication stress [7], treatment with TH588 or TH1579 does not result in incorporation of oxidized nucleotides during the S phase of the cell cycle [5]. Even more surprising is that when replacing the normal DNA polymerase δ (PolD) with an error-prone variant that also readily incorporates 8-oxodGTP, no replication stress or incorporation of oxidized nucleotides are observed in the S phase of the cell cycle [5]. In contrast, injection of 8-oxodGTP is toxic to cells when treated with TH588, TH1579 [8] or an MTH1 inhibitor that is normally not toxic [4]. Altogether, this suggests that oxidative stress levels are too low in S-phase cells to generate 8-oxodGTP, and also in cancer cells under oncogene pressure. This is further supported by ROS being generated in cells under prolonged mitotic arrest, resulting in mitophagy causing ROS [9]. A model is emerging where TH588 or TH1579 arrest cancer cells in mitosis by disrupting microtubule polymerization (likely also independently of MTH1 [3]) (Figure S1). It is unclear why TH588 and TH1579 do not perturb microtubule polymerization in non-transformed cells, which are also not arrested in mitosis [4]. The mitotic arrest results in ROS accumulation, damaging the nucleotide pool generating 8-oxodGTPs, which in the presence of TH588 or TH1579 are incorporated during mitotic DNA synthesis (MIDAS), also contributing to cell death [5] (Figure S1). Mitotic replication (MIDAS) is essentially a repair synthesis process of unrepaired replication stress S-phase lesions that when carried over to G2/M are repaired by processes such as homologous recombination [10,11]. The objective in this study is to understand the molecular reason(s) by which TH588 and TH1579 can kill cancer but not non-transformed cells. Our hypothesis is that cancer cells are sensitive to these inhibitors, owing to high levels of oncogene-induced replication stress [12,13], causing DNA lesions to persist into mitosis, resulting in chromosomal instability [14], mitotic arrest, ROS production and incorporating 8-oxodGTP in cancer cells. Supporting this theory are previous reports linking replication stress to oxidative stress, activating DNA damage response in gliomas [15].

Here, we wanted to generate an isogenic system to study the effect of oncogene-induced replication stress on response to TH588 and TH1579. The single oncogene that is amongst those that most efficiently induce DNA replication fork stress is the transcription factor c-Myc [16,17], which drives cancer and is associated with poor prognosis and unfavorable survival in patients with cancer such as renal cancer, urothelial cancer and ovarian cancer [18,19]. The mechanism underlying c-Myc-induced replication stress is complex and involves both the transcriptional and non-transcriptional roles of c-Myc [16,20]. Elevated c-Myc increases the expression of Cdks (e.g., Cdk4) and cyclins (D1/D2/B) [21,22] which trigger progression through G1, likely causing replication stress. Furthermore, c-Myc represses Cdk inhibitors such as p21 and p15^INK4^, preventing the p53 response and allowing replication on damaged DNA [23]. dNTP levels are also increased following c-Myc expression, which likely increases replication fork speed and stress [24]. The individual contributions to generate replication stress by these individual factors are not yet established. Further to this, emerging data demonstrate that R-loop and replication collisions are key events underlying replication stress [25]. Indeed, collisions between replication–transcription complexes, alterations of nucleotide pools or metabolic processes resulting in increased levels of ROS that induce DNA damage are likely all contributing to c-Myc-induced replicative stress [17]. The increased load of ROS observed in cancer can result in direct oxidation of DNA or, preferentially, cause damage within the free dNTP pool. One of the major products of nucleotide oxidation is 8-oxo-2′-deoxyguanosine-triphosphate (8-oxodGTP), that upon mispairing with adenine once incorporated into DNA, results in mutations and cell death.

Here, we decided to study the effect of c-Myc overexpression on sensitivity following TH588 or TH1579 treatment. Our results show that cells overexpressing c-Myc accumulate in the S phase of the cell cycle, have slower replication fork speed and suffer from replication stress. Moreover, these cells are sensitive to TH588 and TH1579, and the levels of c-Myc drastically drop following TH588 or TH1579 treatment. The lost viability could be rescued by the addition of transcriptional, proteasomal and CDK1 inhibitors, most likely via different signaling pathways. This indicates that c-Myc overexpression induces sensitivity to TH588 or TH1579 treatment, which could be used clinically, especially in cancers with known c-Myc deregulation such as aggressive prostate cancer, ovary cancer and breast cancer [26].

## 2. Materials and Methods

### 2.1. Cells and Culture Conditions

Human immortal, non-tumorigenic HA1EB cells were cultured in Dulbecco’s modified Eagle’s medium (Gibco, ThermoFisher, Waltham, MA, USA) supplemented with 10% fetal bovine serum and Pen/Strep in a humidified CO_2_ atmosphere at 37 °C. The c-Myc overexpressing cells and the control cells (empty vector) were generated by stable transfection with PB-GFP or PB-GFP-c-Myc (pHULK piggyBac Mammalian Expression Vectors as previously described [27].

### 2.2. Drug Treatments

Compounds used in this study include: cordycepin (Sigma-Aldrich, St. Louis, MO, USA; cat# C3394), RO-3306 (Sigma-Aldrich; cat# SML0569), Mitomycin C (Sigma-Aldrich; cat# M4287), bortezomib (Selleckchem, Houston, TX, USA; cat# PS-341) and dNTPs (Sigma-Aldrich; cat# U3003, C4654, G6264, A4036). TH588 and TH1579 were synthesized in-house, whereas AZ19 compound was provided by AstraZeneca (Södertälje, Sweden).

### 2.3. Transfection

Cells were grown in an either 6-well or 96-well plate setup with a seeding density of 50,000 cells or 1500 cells per well, respectively. At 24 h after seeding, the cells were transfected using 10 nM siRNA. INTERFERin^®^ (Polyplus, Illkirch-Graffenstaden, France; cat#409-10) was used as transfection reagent. As negative control, AllStars Negative control siRNA (QIAGEN, cat# SI03650318) was used. To avoid starvation in a 96-well plate setup, 24 h after transfection 50 µL serum containing media was added. The following MTH1 siRNA sequence was used: 5′-CGACGACAGCUACUGGUUU-3′ (siMTH1#3).

### 2.4. Antibodies

The following antibodies were used in this study: mouse anti-β-actin (Abcam, Cambridge, UK; cat# ab6276), mouse anti-γH2AX-S139 (Millipore, Burlington, MA, USA; cat# 05-636), mouse anti-c-Myc (Santa Cruz, Dallas, TX, USA; cat# sc-42), rabbit anti-cleaved PARP Asp214 (Cell Signaling, Danvers, MA, USA; cat# 9541), rabbit anti-MTH1 (Novus Biologicals, Centennial, CO, USA; cat# NB100-109), rabbit anti-p53 pS15 (Cell Signaling; cat# 9284), mouse anti-p53 (Santa Cruz; cat# sc-126), mouse anti-GAPDH (Abcam; cat# ab8245), rat anti-RPA32 (Cell Signaling; cat# 2208).

The secondary antibodies used were: goat anti-rat Alexa Fluor^®^ 568 (Life Technologies, Carlsbad, CA, USA; cat# A-11077), goat anti-rat Alexa Fluor^®^ 647 (Life Technologies; cat# A-21247), IRDye^®^ 800CW donkey anti-rabbit (LI-COR, Lincoln, NE, USA; cat# 926-32213), IRDye^®^ 680RD donkey anti-rabbit (LI-COR; cat# 926-68073), IRDye^®^ 800CW donkey anti-mouse (LI-COR; cat# 926-32212), IRDye^®^ 680RD donkey anti-mouse (LI-COR; cat# 926-68072).

### 2.5. DNA Fiber Analysis

HA1EB cells were pulse-labeled with 25 μM CldU for 20 min, washed with medium and pulse-labeled with 250 μM IdU for 30 min. Labeled cells were harvested and DNA fiber spreads were prepared as described elsewhere [28]. CldU was detected by incubating acid-treated fiber spreads with rat anti-BrdU monoclonal antibody (Abcam; cat# ab6326), whereas IdU was detected using mouse anti-BrdU monoclonal antibody (BD Biosciences, San Jose, CA, USA; cat# 347580) for 2.5 h at RT. Slides were fixed with 4% PFA and incubated with goat anti-rat Alexa Fluor 555 or goat anti-mouse Alexa Fluor 488 for 1.5–2 h. Fibers were examined using a Zeiss (Jena, Germany) LSM710 confocal laser scanning microscope with a 63× oil immersion objective. For quantification of replication structures, at least 250 structures were counted per experiment. The lengths of red-labeled (AF 555) or green-labeled (AF 488) patches were measured using the ImageJ software (National Institutes of Health; http://rsbweb.nih.gov/ij/, accessed on 3 February 2014) and arbitrary length values were converted into micrometers using the scale bars created by the microscope.

### 2.6. EdU Incorporation

Cells were incubated in media supplemented with 10 μM EdU for 15 min, washed in PBS and then fixed using 4% PFA, 0.1% Triton-X in PBS at RT for 15 min. EdU incorporation was visualized using Click-iT^®^ EdU Alexa Fluor^®^ 594 Imaging Kit (Life Technologies; cat# C10337) according to manufacturer’s instructions. Cells were counterstained with DAPI before imaging.

### 2.7. Immunofluorescence Microscopy

Cells were grown on coverslips, fixed with 4% PFA for 15 min and permeabilized with 0.1% Triton-X-100 for 5 min. Cells were kept in blocking buffer for 1 h (2% BSA, 5% glycerol, 0.2% Tween20, 0.1% NaN_3_), followed by 1 h incubation in primary antibody and 30 min in secondary antibody. DNA was stained with DAPI and mounted using ProLong^®^ Gold Antifade Mountant (Molecular Probes, Carlsbad, CA, USA; cat# P36934). Imaging was carried out using a Zeiss LSM710 confocal laser scanning microscope and Zen software (2012). For RPA32 immunostainings, cells were pre-extracted with 0.1% Triton X-100 for 30 s prior to fixation. Samples were incubated overnight with rat anti-RPA32 and 1 h with goat anti-rat Alexa Fluor^®^ 647.

### 2.8. Flow Cytometry

Cells were harvested, washed in PBS and fixed in 70% ethanol for 60 min at −20 °C or stored until analyzed. Cells were stained in PBS containing 5 μg/mL 7-AAD (7-amino-actinomycin D), 20 μg/mL RNase A and 0.1% Triton X-100 for 1 h in 4 °C. Cell cycle profiles were analyzed using a Navios flow cytometer (Beckman Coulter, High Wycombe, UK) and Kaluza analysis software (version 1.2).

### 2.9. qRT-PCR

The Direct-zol™ RNA MiniPrep kit (Zymo Research, Irvine, CA, USA; cat#R2052) was used to isolate RNA from cultured cells. cDNA was synthesized using the QuantiTect^®^ Reverse Transcription kit (QIAGEN, Hilden, Germany; cat#205313) with 400 ng RNA as starting material. The iTaq Universal SYBR Green Supermix (BioRad, Hercules, CA, USA; cat#172-5085) was used to perform qRT-PCR with a CFY96 real-time PCR machine (BioRad). Relative expression on mRNA level was calculated in comparison to GAPDH and β-actin. Primer sequences can be found in the Supplementary Material (Table S1).

### 2.10. Western Blotting

Cells were harvested and washed in PBS and proteins were extracted in lysis buffer containing 100 mM Tris-HCL at pH 8, 150 mM NaCl, 1% NP-40 supplemented with phosphatase and protease inhibitors, for 30 min on ice. Protein concentrations were determined using Pierce™ BCA protein assay kit (Thermo Fisher Scientific, Waltham, MA, USA; cat# 23227) and Western blotting was carried out according to standard protocols.

### 2.11. Resazurin Assay

Cells were seeded in 96-well plates (1500 cells/well) and treated with the indicated drugs and doses. Then, 10 μg/mL resazurin was added to the cells for 4 h before analysis using a Hidex Sense microplate reader. The absorbance was normalized against background levels and the data were processed in Microsoft Excel.

### 2.12. Clonogenic Survival Assays

In a 6-well plate setup, 1000 cells/mL were seeded and treated immediately. After 48 h incubation, media containing vehicle or inhibitor were replaced with fresh media and continuously incubated for 5–6 days. Before manual counting of colonies, 4% methylene blue in MeOH was utilized to fix and stain the cells.

### 2.13. Statistical Analysis

Statistical significance was determined via two-tailed Student’s *t*-test using Microsoft Excel. The results originate from at least two independent experiments and are presented as mean ± standard error of the mean (S.E.M).

## 3. Results

### 3.1. Generating and Characterizing c-Myc Overexpressing HA1EB Cells

**Figure 1:**
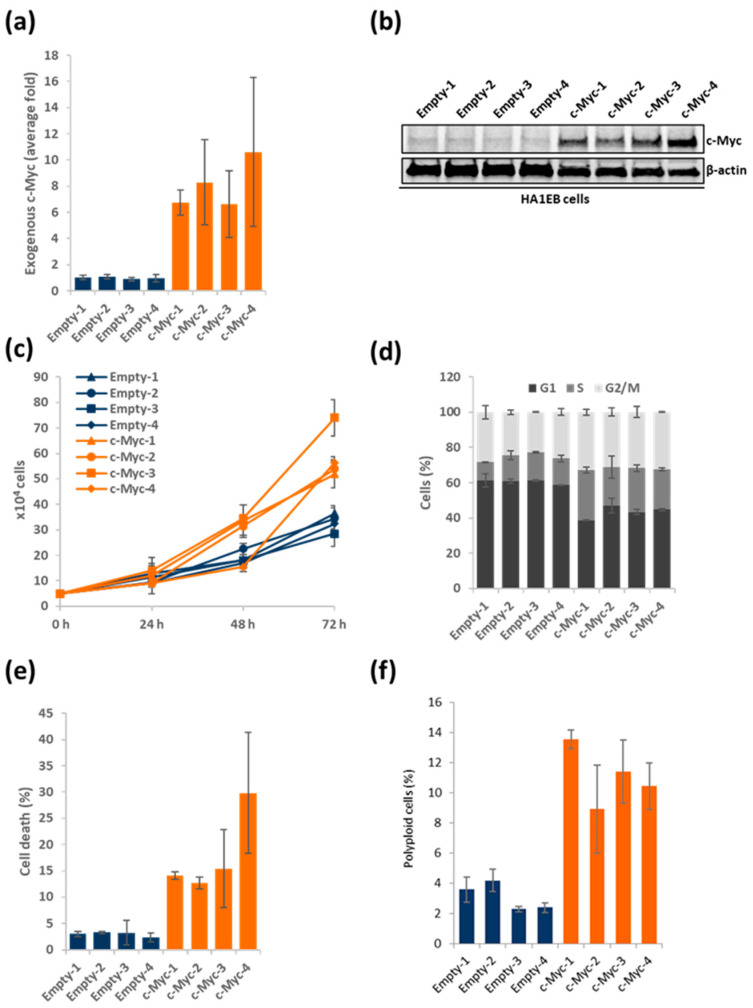
HA1EB cells overexpressing c-Myc proliferate faster. (**a**) Exogenous c-Myc mRNA levels in HA1EB cells, *n* = 3 ± S.E.M. (**b**) Exogenous c-Myc protein expression in HA1EB cells. (**c**) HA1EB cells were seeded and counted daily, *n* = 2 ± S.E.M. (**d**) Cell cycle profiles of HA1EB cells, *n* = 2 ± S.E.M. (**e**,**f**) HA1EB cells were seeded and left to grow for 72 h. Then, cells were harvested, stained with 7-AAD and analyzed using FACS. Dead cells (**e**), <2N DNA content and polyploid cells (**f**), >4N DNA content, *n* = 2 ± S.E.M.

In order to study the direct effects of c-Myc overexpression, we generated HA1EB cell lines that stably expressed full-length c-Myc. HA1EB cells are derived from human epithelial kidney cells, immortalized via SV40 large T antigen and hTERT expression. The main function of SV40 large T is to block the function of both Rb and p53 and hence the cell cycle checkpoint control against cancer growth [29], while the function of hTERT is to allow continuous growth without shortening of telomeres. In general, these cells are considered genetically stable and can only form tumors if they are transformed by an oncogene to promote growth [30,31]. Following GFP-tagged plasmid transfection (empty or c-Myc expressing vectors) as described elsewhere [27], cells were selected by flow cytometry and grown from the single-cell level into several different clones (Figure S2A). The selected clones (termed #1-4) were screened for c-Myc expression compared to the empty vector control. Indeed, all four selected clones carried high c-Myc expression both on mRNA and protein level compared to control cells (Figure 1a,b and Figure S5). Moreover, levels of endogenous c-Myc mRNA were decreased (Figure S2B), which is in line with previous published data, where exogenous c-Myc negatively regulates endogenous c-Myc mRNA expression [32]. As overexpression of c-Myc triggers replication stress and subsequent DNA damage, this would normally activate the cell cycle arrest and apoptotic pathways. Here, the inactivation of the p53 response with SV40 large T [29] will make the cells tolerate a higher c-Myc expression level, as previously demonstrated [30,31], and the overall level of c-Myc in the clones will be balanced to not be too high to induce intolerable replication stress and not too low to provide insufficient growth advantage. This is like how c-Myc levels are also balanced in cancer to promote growth and a result of how much the cell can tolerate.

Next, we studied the mRNA levels of MTH1 in the HA1EB cells and found no significant difference between empty vector and c-Myc overexpressing cells (Figure S2C). However, the known target of c-Myc, cyclin E1, displayed higher mRNA levels following c-Myc overexpression using clones 3 and 4 as representative models (Figure S2D). Altogether, these data prompted us to continue the investigation of these cell lines as models for response following induced c-Myc overexpression.

We started by studying the cellular proliferation rates in the c-Myc overexpressing cell lines compared to their respective controls. The c-Myc overexpressing cells proliferated much faster than control cells, clearly visible following 72 h (Figure 1c). Next, we studied the cell cycle profile of the c-Myc overexpressing cells. All four c-Myc overexpressing clones had less G1 content, whereas the cells in S phase increased compared to the cells expressing empty vectors (Figure 1d). All four c-Myc overexpressing cell lines experienced more endogenous cell death compared to the control cells (Figure 1e). Subsequently, the amount of polyploid cells was determined, and we found that the c-Myc overexpressing cells showed an accumulation of these cells compared to control HA1EB cells (Figure 1f). c-Myc overexpression correlated with cleaved PARP1, phosphorylated p53 and increased γH2AX signaling (Figures S2E and S6), indicating induction of DNA damage and increased cell death upon overexpression of c-Myc alone. As mentioned above, the c-Myc levels tolerated in the overexpressing clones is likely leveled at a balance between growth advantage and cell death.

### 3.2. c-Myc Overexpression Results in EdU and RPA32 Accumulation

**Figure 2:**
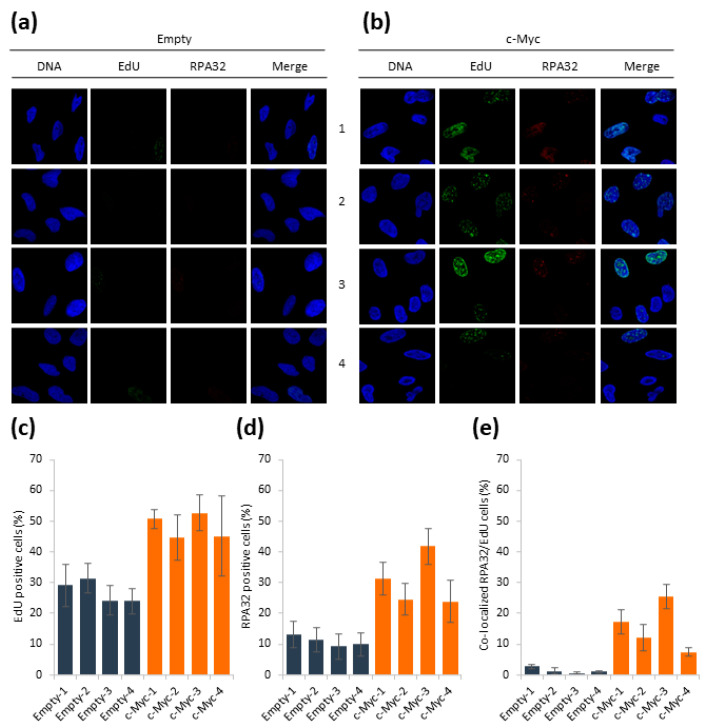
Increased EdU incorporation and RPA32 foci in c-Myc overexpressing HA1EB cells. (**a**,**b**) HA1EB cells were labeled with EdU for 15 min and stained with EdU Click-iT (according to the manufacturer’s instructions) and anti-RPA32 antibody. DNA was stained using DAPI. Images were taken using a Zeiss LSM-780 confocal microscope and analyzed using Image J software. A total of 150 cells/sample was counted, *n* = 3 ± S.E.M. (**c**) Quantification of EdU incorporation. Mean intensity ≥5 AU was considered as positive. (**d**) Percentage of cells with ≥5 RPA32 foci. (**e**) Percentage of cells with ≥5 RPA32/EdU co-localized foci.

Since the c-Myc overexpressing cells accumulated in the S phase, we wanted to study DNA synthesis and the replication response in these cells. First, we measured incorporation of the thymidine analogue 5-ethynyl-2′-deoxyuridine (EdU). As expected by the cell cycle profile, the EdU-positive cell population was higher in the cells overexpressing c-Myc compared to the control cells (Figure 2a–c). Moreover, we could detect more single-stranded DNA (ssDNA) accumulation upon staining cells using RPA32 in the c-Myc overexpressing cells compared to empty control cells (Figure 2a,b,d). Strikingly, the co-localization of EdU and RPA32 was clearly induced in the HA1EB cells overexpressing c-Myc compared to the control cells (Figure 2e). Overall, these data reveal potential replication fork stalling following the visualization of RPA32 accumulation in S-phase cells.

### 3.3. Excess of c-Myc Induces Replication Stress

**Figure 3:**
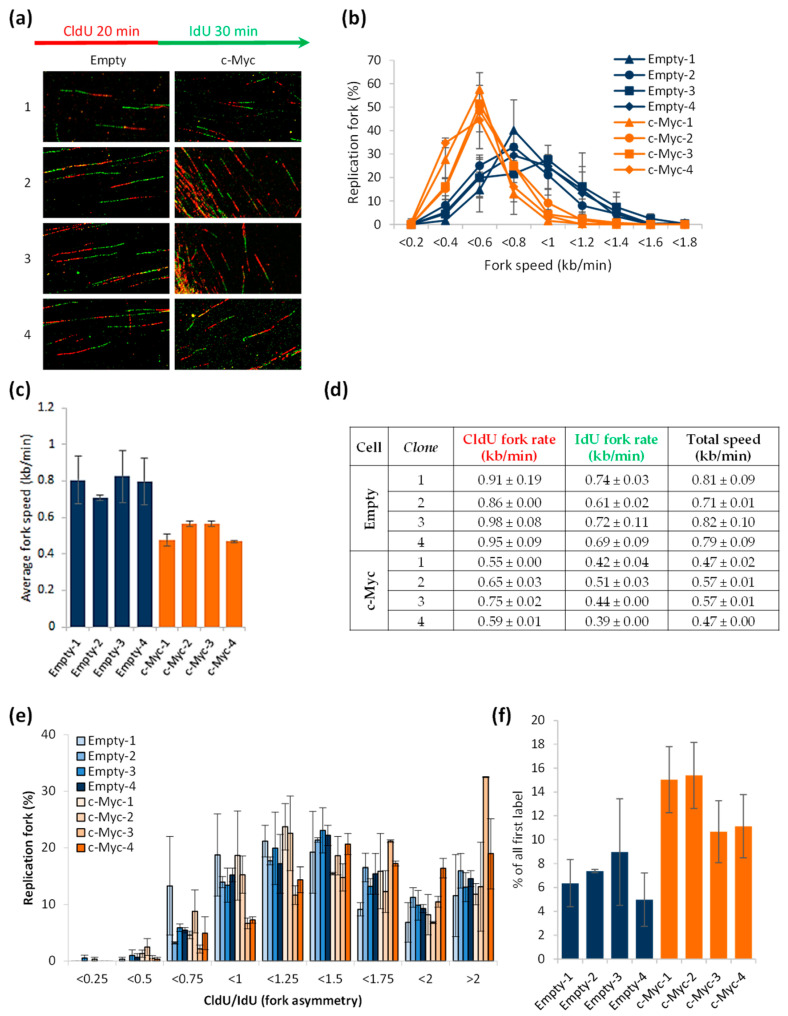
c-Myc overexpression induces replication stress. (**a**–**f**) Cells were seeded and left to grow for 48 h. After that, cells were labeled for 20 min with CldU, washed and labeled with IdU for 30 min. Finally, cells were harvested, and the DNA fibers were obtained. DNA containing CldU was stained in red and DNA containing IdU was stained in green. The length of individual, well-spread labeled fibers was measured and converted into kb/min. Quantitative data presented as means, *n* = 2 ± S.E.M. (**a**) Schematic illustration of CldU (red) and IdU (green) labeling during the assay and representative images of replicative fork tracks for control cells (HA1EB empty) and c-Myc overexpressing cells (HA1EB c-Myc). (**b**) Graph shows the distribution of fork progression speed (kb/min) of the first and second pulses. (**c**) Average of the replication fork extension rates during the first and the second pulses. (**d**) Quantification of the mean replication fork speed (kb/min) during the first (CldU, 20 min), the second (IdU, 30 min) and both pulses. (**e**) Distribution of CldU/IdU ratio of replication fork progression. The value equal to 1 means the extension speed was similar during both pulses (perfect symmetry). (**f**) First-label origins (green–red–green) are shown as percentage of all red (CldU) labeled tracks.

In order to directly test if the HA1EB cells overexpressing c-Myc presented replication stress, we performed the DNA fiber assay. Cells were pulse-labeled with CldU for 20 min, followed by washing and a second pulse of IdU for 30 min (Figure 3a, schematic illustration). Thereafter, labeled cells were harvested and the DNA fiber assay was performed. Indeed, the c-Myc overexpressing cells showed significantly shorter DNA fibers compared to control cells (Figure 3a–d), indicative of replication stress. To determine whether c-Myc overexpression induced fork collapse, we quantified the CldU/IdU ratios. A CldU/IdU value close to 1 means an equivalent fork progression rate during both labeling periods, so perfect fork symmetry, whereas values higher than 2 mean fork asymmetry and fork stalling during the second labeling. The c-Myc overexpressing cells did not show significant differences compared to control cells, with an average CldU/IdU ratio close to 1–1.5 (Figure 3e), except for the c-Myc-3 clone. These results disagree with those obtained in previous publications [20,33], where a higher degree of fork asymmetry in c-Myc overexpressing cells has been observed. This discrepancy could be due to the differences in experimental design, since we grew our cells for months in order to obtain our clones, which could give the cells enough time to adapt to the c-Myc-induced replication stress. Indeed, it is known that c-Myc overexpression is lethal for the cells if they do not develop mechanisms to compensate and reduce the consequences of replication stress as, for example, DNA double-strand breaks [34]. Finally, we quantified the number of firing replication origins. Origins that fired during the first labeling period have continuous IdU-CldU-IdU tracks (green–red–green signal) [35]. It is known that c-Myc overexpression is associated with an excess number of active replication origins to support the acceleration of the S phase [34]. Consequently, c-Myc overexpressing cells showed a higher number of first-label origins than control cells (Figure 3f). Altogether, these results support that HA1EB cells overexpressing c-Myc suffer from replication stress.

### 3.4. TH588 or TH1579 Treatment Decreases Viability of c-Myc Overexpressing Cells

**Figure 4:**
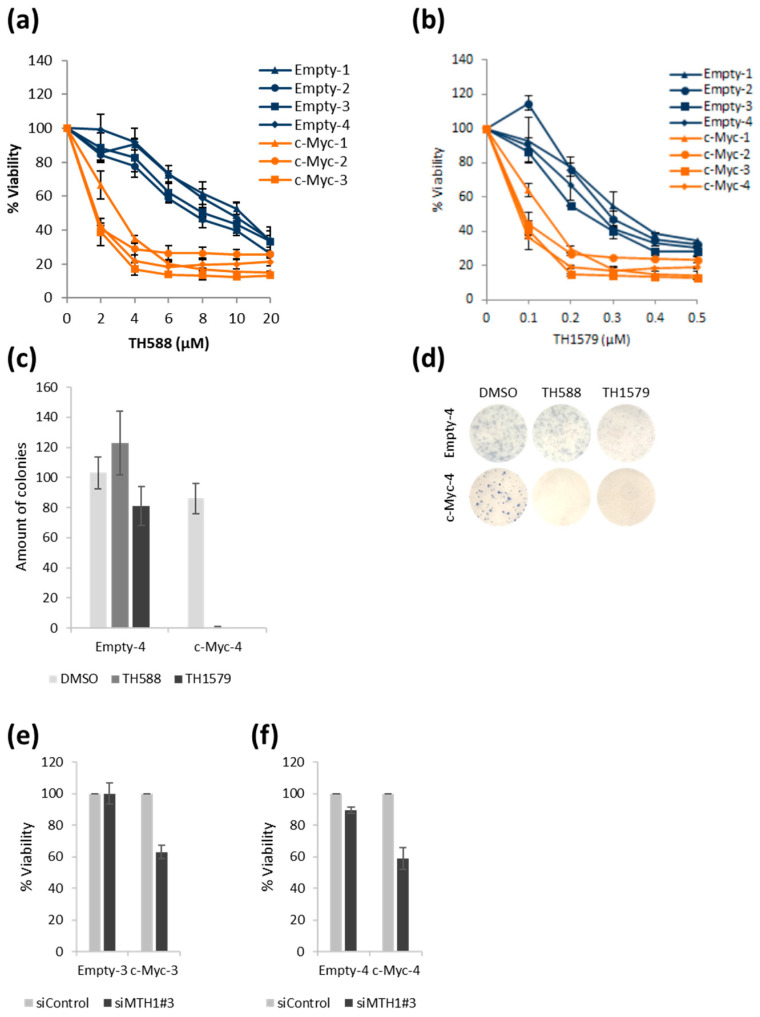
Viability of c-Myc overexpressing cells is decreased upon TH588 or TH1579 treatment or MTH1 silencing. (**a**,**b**) Empty and c-Myc overexpressing cells were plated and treated with the indicated concentrations of TH588 or TH1579 (or DMSO). After 72 h, 10 μg/mL resazurin was added for 4 h and viability was determined (values are normalized to DMSO), *n* = 3 (**a**), *n* = 2 (**b**) ± S.E.M. (**c**) Empty-4 and c-Myc-4 cells were seeded, immediately treated with 5 µM TH588 or 0.5 µM TH1579 for 48 h, new media were added every 2–3 days until colony staining (9–10 days after seeding). The values were normalized to DMSO control, *n* = 2 ± S.E.M. (**d**) Representative clonogenic survival assay images of Empty-4/c-Myc-4 cells. (**e**,**f**) Cells were seeded and transfected with 10 nM siRNA. After 72 h, 10 μg/mL resazurin was added for 4 h and viability was determined (values are normalized to siControl), (**e**) *n* = 3, (**f**) *n* = 2 ± S.E.M.

Having established that our HA1EB cells overexpressing c-Myc displayed the expected phenotype, we decided to test if these cells were sensitive to TH588 or TH1579 treatment. We treated all eight cell lines with varying concentrations of mitotic MTH1 inhibitors, TH588 (Figure 4a) and TH1579 (Figure 4b). Following 72 h, both mitotic MTH1 inhibitors showed higher cytotoxicity against c-Myc overexpressing cells than control cells (Figure 4a,b). Next, we decided to scale down the experiments and focus on two different cell lines, clones 3 and 4, used in the clonogenic survival experiments of control and c-Myc overexpressing cells following mitotic MTH1 inhibitor treatment. The c-Myc-4 overexpressing cells displayed a clear decrease in surviving clones, going down to zero colonies following mitotic MTH1 inhibitor treatment (Figure 4c,d). Conversely, the control cells were basically unaffected at the concentration used (Figure 4c,d). Similar results were found in c-Myc-3 cells (data not shown). Finally, we depleted MTH1 using siRNA in order to see if the observed toxic effect was specific to the mitotic MTH1 inhibitors or not. The knockdown appeared to function well following 72 h (Figures S3A and S7). At that timepoint, the viability of c-Myc overexpressing cells was also decreased (Figure 4e,f), but not as intensely as compared to the mitotic MTH1 inhibitors.

### 3.5. Mitotic MTH1 Inhibitors TH588 and TH1579 Deplete c-Myc Protein and Cause Cell Death

**Figure 5:**
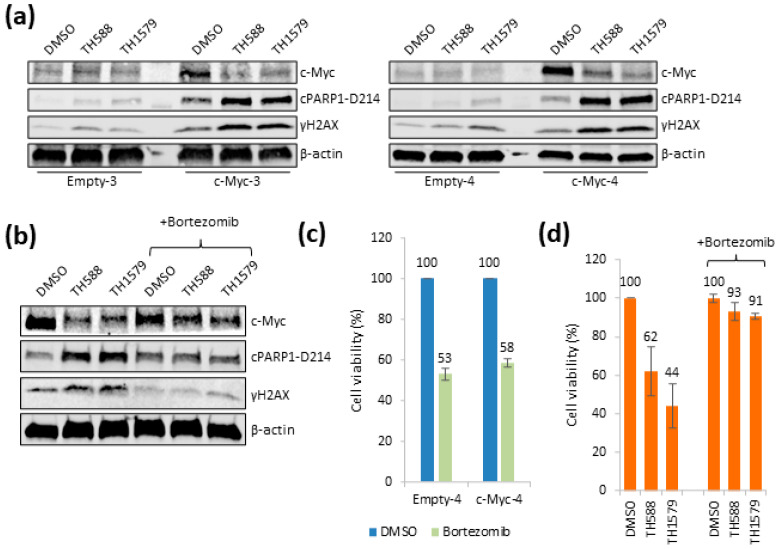
TH588 and TH1579 trigger cell death by degradation of c-Myc protein. (**a**) Empty-3/c-Myc-3 or Empty-4/c-Myc-4 were treated with 5 µM TH588 or 0.5 µM TH1579 for 24 h. Following cell lysis, the indicated proteins were blotted. (**b**) c-Myc-4 cells were treated with 5 µM TH588 or 0.5 µM TH1579 for 24 h ± co-addition of bortezomib (10 nM). The indicated proteins were blotted. (**c**,**d**) c-Myc-4 cells were seeded and following 24 h, the cells were treated with 5 µM TH588 or 0.5 µM TH1579 for 24 h ± co-addition of bortezomib (10 nM). Then, 10 μg/mL resazurin was added for 4 h and viability was determined (values are normalized to internal DMSO control), *n* = 2 ± S.E.M.

In order to determine the toxic mechanism of action of TH588 or TH1579 in c-Myc overexpressing cells, we studied the expression of c-Myc, cleaved PARP1 and γH2AX following 24 h exposure to TH588 or TH1579. Surprisingly, c-Myc levels drastically dropped following TH588 or TH1579 treatment, which correlated with cleaved PARP1 and γH2AX in the c-Myc overexpressing cells (Figure 5a and Figure S10). This effect overlaps with the reduced survival of cells following TH588 or TH1579 treatment in c-Myc overexpressing cells. Next, we studied the effect on the c-Myc mRNA expression following TH588 or TH1579 treatment but found no significant difference following 24 h of treatment (Figure S3B). This shows that TH588 and TH1579 only negatively affect c-Myc protein levels and not mRNA levels. After this, we decided to test a non-toxic MTH1 inhibitor, AZ19, originally produced at AstraZeneca [36], to determine its effect on c-Myc levels. We also included mitomycin C (MMC), previously published to reduce c-Myc protein expression levels [37]. Interestingly, the non-toxic MTH1 inhibitors did not affect c-Myc levels nor induced γH2AX or PARP1 cleavage (Figures S3C and S8). MMC gave similar c-Myc reduction as TH588 and TH1579, and increased activation of γH2AX and cleaved PARP1 (Figures S3C and S8). Next, we silenced MTH1 expression to determine whether c-Myc levels also decreased using this condition. Although we observed a decreased survival of c-Myc overexpressing cells following MTH1 silencing, we did not observe any drop in c-Myc levels (Figures S3D,E and S9) following MTH1 knockdown. However, γH2AX was induced and PARP1 cleaved, whereas c-Myc levels appeared to increase upon exposure to MTH1 siRNA. This suggests that loss of MTH1 from cells via RNAi induces reduced cell viability, but not via destabilization of c-Myc protein levels.

Since neither TH588 nor TH1579 affected c-Myc transcription, we reasoned that the treatment may trigger proteolytic degradation of c-Myc. To test this, we used the proteasome inhibitor bortezomib which showed some stabilization of c-Myc following TH588 or TH1579 treatment (Figure 5b and Figure S11), which also correlated with no increase in cPARP1 and γH2AX. Following this, we wanted to determine if the lack of c-Myc loss caused by bortezomib also increased the viability. While bortezomib treatment itself was the same in c-Myc overexpressing cells compared to control cells (Figure 5c), we found that co-treatment with bortezomib rescued toxicity induced by TH588 and TH1579 (Figure 5d). These data suggest that one mechanism by which TH588 and TH1579 kill c-Myc overexpressing cells is by degradation of c-Myc itself.

### 3.6. Transcription and CDK1 Inhibition Reverse Reduced Viability of Mitotic MTH1 Inhibitors TH588 and TH1579 in c-Myc Overexpressing Cells

**Figure 6:**
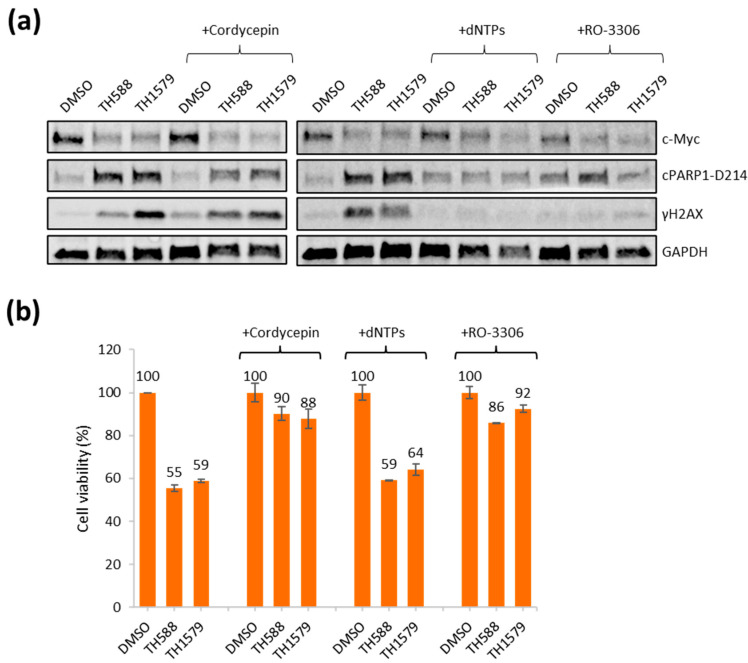
Inhibition of transcription and CDK activity reverse DNA damage and toxicity of mitotic MTH1 inhibitors TH588 and TH1579 in c-Myc overexpressing cells. (**a**) c-Myc-4 cells were treated with 5 µM TH588 or 0.5 µM TH1579 for 24 h ± co-addition of cordycepin (50 µM), nucleosides (dNTPs) (50 µM) or RO-3306 (5 µM). The indicated proteins were blotted. (**b**) c-Myc-4 cells were seeded and following 24 h, the cells were treated with 5 µM TH588 or 0.5 µM TH1579 for 24 h ± co-addition of cordycepin (50 µM), nucleosides (dN) (50 µM) or RO-3306 (5 µM). Then, 10 μg/mL resazurin was added for 4 h and viability was determined (values are normalized to internal DMSO control), *n* = 2 ± S.E.M.

Here, we established isogenic c-Myc overexpressing cells that have high levels of replication stress. Next, we aimed at determining if we were able to rescue the toxic effects of TH588 or TH1579 treatment in the c-Myc overexpressing cells by reversing replication stress. We and others have demonstrated that replication stress can be reversed by inhibition of CDK activity [38], inhibition of transcription using cordycepin [39] or addition of nucleosides [14,40]. Here, we used the CDK1 inhibitor RO-3306, cordycepin and nucleoside rescue for 24 h and monitored c-Myc levels, DNA damage by γH2AX and apoptosis-cleaved PARP1 using Western Blotting (Figure 6a and Figure S12). First, we explored the toxicity of the selected agents, and most of them were more toxic to the c-Myc overexpressing cells compared to control cells, except dNTPs (Figure S4). We observed no difference in c-Myc levels but reduced levels of both cleaved PARP1 and γH2AX upon addition of nucleosides and RO-3306, while the drop in DNA damage (γH2AX) was modest with cordycepin (Figure 6a and Figure S12). The loss of the DNA damage (γH2AX) marker is a clear signal of reduced replication stress, in particular following RO-3306 and nucleoside treatment. In line with the hypothesis that replication stress is related to TH588- and TH1579-induced toxicity in Myc overexpressing cells, we observed that both cordycepin and RO-3306 reversed TH588- and TH1579-induced toxicity (Figure 6b). This is also in line with the observed reduced levels of cleaved PARP and γH2AX following MTH1 inhibition. Nucleoside addition did not rescue reduced viability after TH588 or TH1579 treatment (Figure 6b), which is likely related to that c-Myc expression induces dNTP [24] and that replication stress in these cells may not be related to reduced dNTP levels.

## 4. Discussion

In this study, we generated isogenic HA1EB cells overexpressing c-Myc that displayed the expected phenotype associated with oncogene overexpression, i.e., faster proliferation, increased cell death, S-phase accumulation and DNA replication stress. This all points to that the cell system is ideally suited to study the effect of c-Myc. The c-Myc level obtained is likely a result of c-Myc-expression-induced death and growth. Indeed, we see both cell death and growth are increased (Figure 1), and the obtained levels are likely at the balance where there are growth advantages and acceptable death rates. We used this new cell model to determine if c-Myc overexpression is sensitizing these cells to TH588 and TH1579, as previously shown with the Ras oncogene [1]. Indeed, we find that c-Myc overexpressing cells are more sensitive to TH588 and TH1579, accompanied with γH2AX induction and PARP1 cleavage, which would be in line with DNA damage induction and cellular apoptosis previously reported after TH588 or TH1579 treatment.

The second aim was to understand the molecular mechanism sensitizing c-Myc overexpressing cells, and we found to our surprise that the levels of c-Myc dropped upon TH588 and TH1579 treatment. Since c-Myc mRNA levels were unchanged and a proteasomal inhibitor bortezomib prevented c-Myc degradation, we conclude that TH588 and TH1579 treatment triggers proteasomal degradation of c-Myc. Furthermore, since bortezomib decreases TH588 and TH1579 cell killing, we propose that this proteasomal targeting of c-Myc is the contributing mechanism to killing these cells. Since c-Myc is considered to be an undruggable target due to its essential cellular functions, it is interesting to identify compounds that indirectly target c-Myc. Although we do not know the exact mechanism(s) as to how these inhibitors negatively regulate c-Myc, this would be of particular interest for future studies including the status of the Myc/Max complex and other means of destabilizing Myc.

Here, we argue that c-Myc proteolytic degradation by TH588 and TH1579 is unrelated to MTH1, as neither MTH1 siRNA nor the structurally unrelated AZ19 MTH1 inhibitor decreases c-Myc levels. It is established that the TH588 and TH1579 compounds have a dual mechanism targeting MTH1 as well as a direct effect on tubulin [3], and this is an effect likely related to any tubulin effect of the compounds, as it also previously has been reported that anti-microtubule drugs result in reduced c-Myc levels [33].

Our original hypothesis was that selective toxicity in cancer cells by TH588 and TH1579 is related to elevated replication stress in cancer, leading to toxic 8-oxodGTP incorporation during mitotic DNA synthesis (MIDAS) (Figure S1). Replication stress is often a consequence of collision between transcription and replication [41], and the transcription inhibitor cordycepin reverses replication stress. Here, we find that cordycepin reverses toxicity by TH1579, but it is not clear that this is owing to the loss of replication stress, as the reduction in γH2AX is only modest. We have also shown that replication stress is reversed by CDK inhibitors [38], and in this case we see both reversed toxicity and reduced DNA damage in line with the hypothesis that replication stress causes toxicity in cancer cells. Although nucleoside supplementation reduces γH2AX induced by TH588 and TH1579 (Figure 6A), it does not reverse TH588- nor TH1579-induced toxicity. As c-Myc increases dNTP pools [24] rather than reducing dNTP pools in the case of cyclin E overexpression [40], one would not expect this to reverse replication stress and toxicity. However, unbalanced dNTP pools may cause asymmetric forks and replication stress, which nucleoside supplementation may improve. However, we did not observe any asymmetric forks following c-Myc expression, so it is unlikely that the nucleoside supplementation would affect this in our cells. Overall, the results obtained here are in line with the original hypothesis, but there is more work to be conducted to establish the definite answer to the mechanism of action.

In conclusion, we have in this study found that oncogenic c-Myc expression functions as a sensitizer to TH588 and TH1579 treatment. We generated an isogenic cell system to have an isogenic model to single out the effect of c-Myc. Despite this, we find that the molecular toxicological mechanism specifically sensitizing c-Myc overexpressing cells to TH588 and TH1579 is complex and involves proteasomal degradation of c-Myc, as well as being dependent on c-Myc transcription and CDK activation. While this study reveals a complex toxic mechanism of TH588 and TH1579 killing c-Myc overexpressing cells, which also involves MTH1-independent effects of the compounds, the cancer selective killing of these compounds is confirmed and supports further development of TH1579 in the clinic in treatment of cancer.

## Data Availability

Not applicable.

## Supplementary Materials

The following supporting information can be downloaded at: https://www.mdpi.com/article/10.3390/biom12121777/s1. Table S1: Primers used in qRT-PCR analysis; Figure S1: Proposed mechanism of TH588 and TH1579 to selectively kill cancer cells; Figure S2: Schematic illustration of cell line generation and marker levels in c-Myc HA1EB cells; Figure S3: Non-toxic MTH1 inhibitors or MTH1 silencing do not decrease c-Myc levels; Figure S4: Viability of used inhibitors in Empty-4 and c-Myc-4 HA1EB cell lines. Figure S5. Uncropped Western blots for Figure 1b. Figure S6. Uncropped Western blots for Figure S2e. Figure S7. Uncropped Western blots for Figure S3a. Figure S8. Uncropped Western blots for figure S3c. Figure S9. Representative uncropped Western blots for figure S3d–e. Figure S10. Representative uncropped Western blots for Figure 5a. Figure S11. Uncropped Western blots for Figure 5b. Figure S12. Uncropped Western blots for Figure 6a.

## References

[1] Gad H., Koolmeister T., Jemth A.-S., Eshtad S., Jacques S.A., Ström C.E., Svensson L.M., Schultz N., Lundbäck T., Einarsdottir B.O. (2014). MTH1 inhibition eradicates cancer by preventing sanitation of the dNTP pool. Nature.

[2] Berglund U.W., Sanjiv K., Gad H., Kalderén C., Koolmeister T., Pham T., Gokturk C., Jafari R., Maddalo G., Seashore-Ludlow B. (2016). Validation and development of MTH1 inhibitors for treatment of cancer. Ann. Oncol..

[3] Kawamura T., Kawatani M., Muroi M., Kondoh Y., Futamura Y., Aono H., Tanaka M., Honda K., Osada H. (2016). Proteomic profiling of small-molecule inhibitors reveals dispensability of MTH1 for cancer cell survival. Sci. Rep..

[4] Gad H., Mortusewicz O., Rudd S.G., Stolz A., Amaral N., Brautigham L. (2019). MTH1 promotes mitotic progression to avoid oxidative DNA damage in cancer cells. bioRxiv.

[5] Rudd S.G., Gad H., Sanjiv K., Amaral N., Hagenkort A., Groth P., Ström C.E., Mortusewicz O., Berglund U.W., Helleday T. (2020). MTH1 Inhibitor TH588 Disturbs Mitotic Progression and Induces Mitosis-Dependent Accumulation of Genomic 8-oxodG. Cancer Res..

[6] Tsuzuki T., Egashira A., Igarashi H., Iwakuma T., Nakatsuru Y., Tominaga Y., Kawate H., Nakao K., Nakamura K., Ide F. (2001). Spontaneous tumorigenesis in mice defective in the MTH1 gene encoding 8-oxo-dGTPase. Proc. Natl. Acad. Sci. USA.

[7] Di Micco R., Fumagalli M., Cicalese A., Piccinin S., Gasparini P., Luise C., Schurra C., Garré M., Giovanni Nuciforo P., Bensimon A. (2006). Oncogene-induced senescence is a DNA damage response triggered by DNA hyper-replication. Nature.

[8] Bräutigam L., Pudelko L., Jemth A.-S., Gad H., Narwal M., Gustafsson R., Karsten S., Puigvert J.C., Homan E., Berndt C. (2016). Hypoxic Signaling and the Cellular Redox Tumor Environment Determine Sensitivity to MTH1 Inhibition. Cancer Res..

[9] Doménech E., Maestre C., Esteban-Martínez L., Partida D., Pascual R., Fernández-Miranda G., Seco E., Campos-Olivas R., Pérez M., Megias D. (2015). AMPK and PFKFB3 mediate glycolysis and survival in response to mitophagy during mitotic arrest. Nat. Cell Biol..

[10] Minocherhomji S., Ying S., Bjerregaard V.A., Bursomanno S., Aleliunaite A., Wu W., Mankouri H., Shen H., Liu Y., Hickson I.D. (2015). Replication stress activates DNA repair synthesis in mitosis. Nature.

[11] Beucher A., Birraux J., Tchouandong L., Barton O., Shibata A., Conrad S., Goodarzi A.A., Krempler A., Jeggo P.A., Lobrich M. (2009). ATM and Artemis promote homologous recombination of radiation-induced DNA double-strand breaks in G_2_. EMBO J..

[12] Bartkova J., Rezaei N., Liontos M., Karakaidos P., Kletsas D., Issaeva N., Vassiliou L.-V.F., Kolettas E., Niforou K., Zoumpourlis V.C. (2006). Oncogene-induced senescence is part of the tumorigenesis barrier imposed by DNA damage checkpoints. Nature.

[13] Halazonetis T.D., Gorgoulis V.G., Bartek J. (2008). An Oncogene-Induced DNA Damage Model for Cancer Development. Science.

[14] Burrell R.A., McClelland S.E., Endesfelder D., Groth P., Weller M.-C., Shaikh N., Domingo E., Kanu N., Dewhurst S.M., Gronroos E. (2013). Replication stress links structural and numerical cancer chromosomal instability. Nature.

[15] Bartkova J., Hamerlik P., Stockhausen M.-T., Ehrmann J., Hlobilkova A., Laursen H., Kalita O., Kolar Z., Poulsen H.S., Broholm H. (2010). Replication stress and oxidative damage contribute to aberrant constitutive activation of DNA damage signalling in human gliomas. Oncogene.

[16] Dominguez-Sola D., Ying C.Y., Grandori C., Ruggiero L., Chen B., Li M., Galloway D.A., Gu W., Gautier J., Dalla-Favera R. (2007). Non-transcriptional control of DNA replication by c-Myc. Nature.

[17] Murga M., Campaner S., Lopez-Contreras A.J., Toledo L.I., Soria R., Montaña M.F., Artista L.D., Schleker T., Guerra C., Garcia E.O. (2011). Exploiting oncogene-induced replicative stress for the selective killing of Myc-driven tumors. Nat. Struct. Mol. Biol..

[18] Chen H., Liu H., Qing G. (2018). Targeting oncogenic Myc as a strategy for cancer treatment. Signal Transduct. Target. Ther..

[19] Miller D.M., Thomas S.D., Islam A., Muench D., Sedoris K. (2012). c-Myc and Cancer Metabolism. Clin. Cancer Res..

[20] Maya-Mendoza A., Ostrakova J., Kosar M., Hall A., Duskova P., Mistrik M., Merchut-Maya J.M., Hodny Z., Bartkova J., Christensen C. (2015). Myc and Ras oncogenes engage different energy metabolism programs and evoke distinct patterns of oxidative and DNA replication stress. Mol. Oncol..

[21] Bouchard C., Thieke K., Maier A., Saffrich R., Hanley-Hyde J., Ansorge W., Reed S., Sicinski P., Bartek J., Eilers M. (1999). Direct induction of cyclin D2 by Myc contributes to cell cycle progression and sequestration of p27. EMBO J..

[22] Rohban S., Campaner S. (2015). Myc induced replicative stress response: How to cope with it and exploit it. Biochim. Biophys. Acta.

[23] Seoane J., Le H.-V., Massague J. (2002). Myc suppression of the p21Cip1 Cdk inhibitor influences the outcome of the p53 response to DNA damage. Nature.

[24] Mannava S., Grachtchouk V., Wheeler L.J., Im M., Zhuang D., Slavina E.G., Mathews C.K., Shewach D.S., Nikiforov M.A. (2008). Direct role of nucleotide metabolism in C-MYC-dependent proliferation of melanoma cells. Cell Cycle.

[25] Kotsantis P., Petermann E., Boulton S.J. (2018). Mechanisms of Oncogene-Induced Replication Stress: Jigsaw Falling into Place. Cancer Discov..

[26] Kalkat M., De Melo J., Hickman K.A., Lourenco C., Redel C., Resetca D., Tamachi A., Tu W.B., Penn L.Z. (2017). MYC Deregulation in Primary Human Cancers. Genes.

[27] Sanjiv K., Hagenkort A., Calderón-Montaño J.M., Koolmeister T., Reaper P.M., Mortusewicz O., Jacques S.A., Kuiper R.V., Schultz N., Scobie M. (2016). Cancer-Specific Synthetic Lethality between ATR and CHK1 Kinase Activities. Cell Rep..

[28] Petermann E., Helleday T., Caldecott K.W. (2008). Claspin Promotes Normal Replication Fork Rates in Human Cells. Mol. Biol. Cell.

[29] Pipas J.M., Levine A.J. (2001). Role of T antigen interactions with p53 in tumorigenesis. Semin. Cancer Biol..

[30] Hahn W.C., Counter C.M., Lundberg A.S., Beijersbergen R.L., Brooks M.W., Weinberg R.A. (1999). Creation of human tumour cells with defined genetic elements. Nature.

[31] Zimonjic D., Brooks M.W., Popescu N., Weinberg R.A., Hahn W.C. (2001). Derivation of human tumor cells in vitro without widespread genomic instability. Cancer Res..

[32] Grignani F., Lombardi L., Inghirami G., Sternas L., Cechova K., Dalla-Favera R. (1990). Negative autoregulation of c-myc gene expression is inactivated in transformed cells. EMBO J..

[33] Puccetti M.V., Adams C.M., Kushinsky S., Eischen C.M. (2019). Smarcal1 and Zranb3 Protect Replication Forks from Myc-Induced DNA Replication Stress. Cancer Res..

[34] Campaner S., Amati B. (2012). Two sides of the Myc-induced DNA damage response: From tumor suppression to tumor maintenance. Cell Div..

[35] Petermann E., Orta M.L., Issaeva N., Schultz N., Helleday T. (2010). Hydroxyurea-Stalled Replication Forks Become Progressively Inactivated and Require Two Different RAD51-Mediated Pathways for Restart and Repair. Mol. Cell.

[36] Kettle J.G., Alwan H., Bista M., Breed J., Davies N.L., Eckersley K., Fillery S., Foote K.M., Goodwin L., Jones D.R. (2016). Potent and Selective Inhibitors of MTH1 Probe Its Role in Cancer Cell Survival. J. Med. Chem..

[37] Frenzel A., Zirath H., Vita M., Albihn A., Henriksson M.A. (2011). Identification of Cytotoxic Drugs That Selectively Target Tumor Cells with MYC Overexpression. PLoS ONE.

[38] Petermann E., Woodcock M., Helleday T. (2010). Chk1 promotes replication fork progression by controlling replication initiation. Proc. Natl. Acad. Sci. USA.

[39] Jones R.M., Mortusewicz O., Afzal I., Lorvellec M., García P., Helleday T., Petermann E. (2012). Increased replication initiation and conflicts with transcription underlie Cyclin E-induced replication stress. Oncogene.

[40] Bester A.C., Roniger M., Oren Y.S., Im M.M., Sarni D., Chaoat M., Bensimon A., Zamir G., Shewach D.S., Kerem B. (2011). Nucleotide Deficiency Promotes Genomic Instability in Early Stages of Cancer Development. Cell.

[41] Maya-Mendoza A., Moudry P., Merchut-Maya J.M., Lee M., Strauss R., Bartek J. (2018). High speed of fork progression induces DNA replication stress and genomic instability. Nature.

