# Supplementary material for: Overexpressed c-Myc Sensitizes Cells to TH1579, a Mitotic Arrest and Oxidative DNA Damage Inducer
Source: Biomolecules. 2022 Nov 29;12(12):1777. doi: 10.3390/biom12121777 (PMC9775511; doi:10.3390/biom12121777)
Supplement: Supplementary file 1 [file biomolecules-12-01777-s001.zip › biomolecules-1893559-supplementary-11.30 (1).pdf]

Supplementary Table S1. Primers used in qRT-PCR analysis.

Target Gene	F/R	Target Sequence	Reference
Exogenous c-Myc	CMYC_F2	TCGGATTCTCTGCTCTCCT	IDT, Ref. No. 72670694/5
	CYCN_R2	CCTCATCTTCTTGTTCCTCCTC	
Endogenous c-Myc	CMYC_F1	GCTGCTTAGACGCTGGATTT	IDT, Ref. No. 72670696/7
	CMYC_R1	GAGTCGTAGTCGAGGTCATAGTT	
Cyclin E	CCNE_F1	GTCCTGGCTGAATGTATACATGC	Sherr, C.J., Cell, 73, 1059-1065 (1993)
	CCNE_R1	CCCTATTTTGTTCAGACAACATGGC	
MTH1	MTH1_F1	AAAGTGCAAGAAGGAGAGACC	IDT, Ref. No. 72670688/9
	MTH1_R1	CTCGCCCACGAACTCAAA	
GAPDH	hGAPDH_F	AAGGTCGGAGTCAACGGATT	M.Altun (Hassink et al., 2009)
	hGAPDH_R	CTCCTGGAAGATGGTGATGG	
β-Actin	hb-actin_F	CCTGGCACCCAGCACAAT	M.Altun (Hassink et al., 2009)
	hb-actin_R	GGGCCGGACTCGTCATACT	

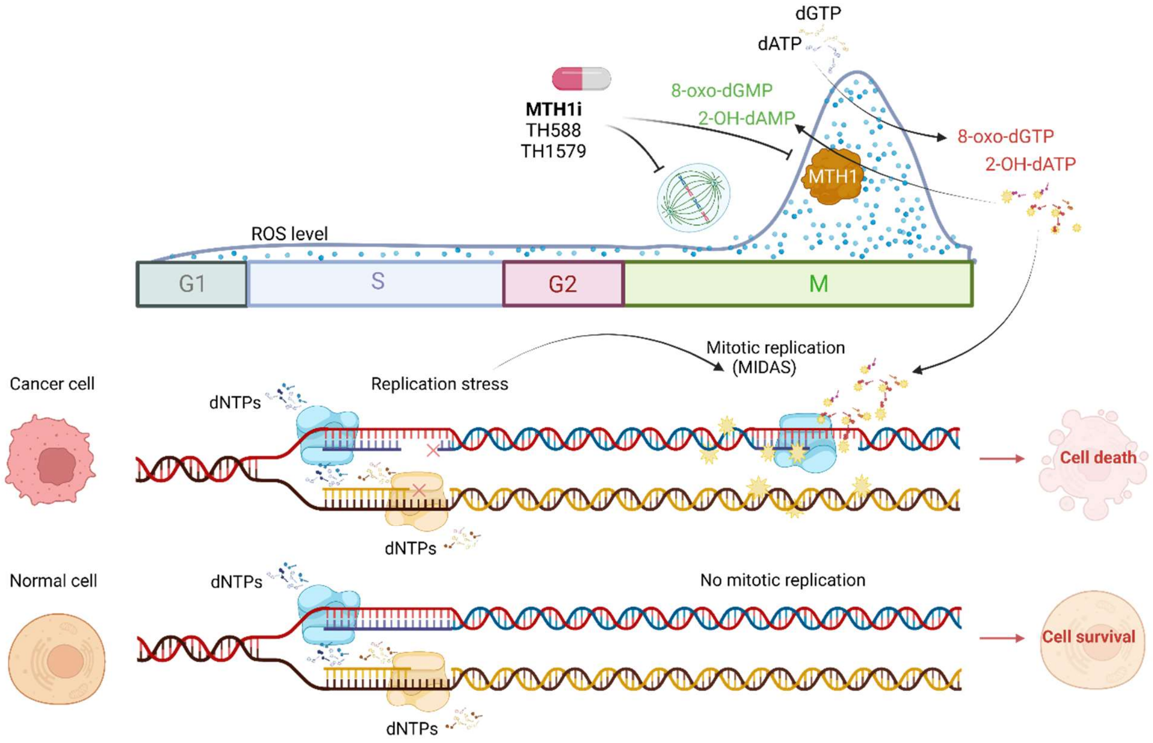

Figure S1. Proposed mechanism of TH588 and TH1579 to selectively kill cancer cells. TH588/TH1579 arrest cells in mitosis which result in accumulation of ROS, oxidizing the dNTP pool in mitosis. Oxidized 8-oxo-dGTP and 2-OH-dATP are degraded by MTH1 to prevent damage incorporation. TH588/TH1579 inhibits MTH1 resulting in damage incorporation of 8-oxodGTP into DNA, leading

to cell death. Here, we hypothesize that unrepaired lesions in S phase, caused by replication stress in cancer, are repaired by mitotic replication (MIDAS). This is cancer specific as normal cells have limited replication stress and no or very little mitotic replication.

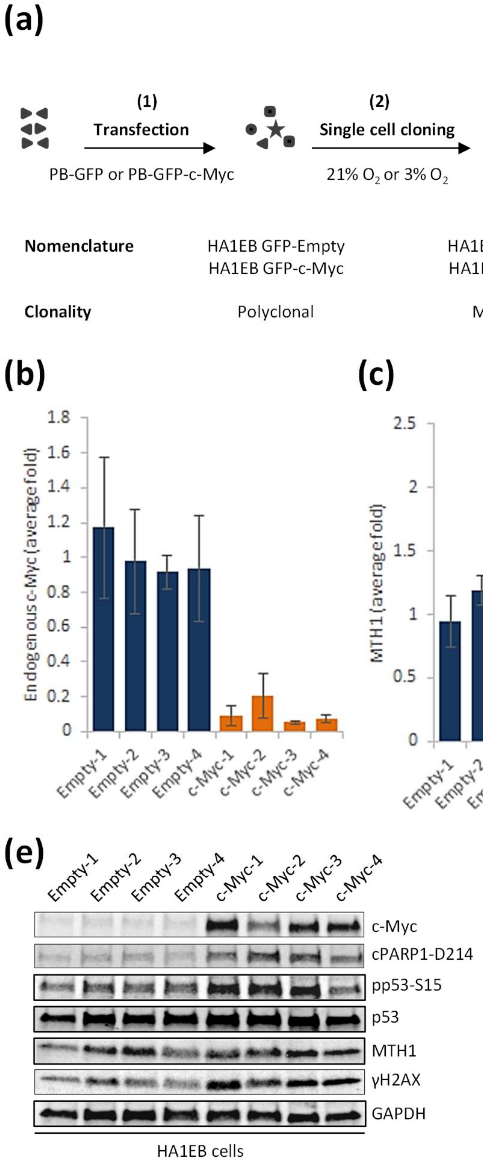

Figure S2. Schematic illustration of cell line generation and marker levels in c-Myc HA1EB cells. (**A**) After transfection with PB-GFP vector (encoding GFP) or PB-GFP-c-Myc vector (encoding GFP and c-Myc), GFP positive cells were selected by FACS (1). Then, cells were seeded to obtain single-cell clones and incubated in normoxia (21% O_2) or hypoxia (3% O_2) (2). Single-cell clones derived from the polyclonal population were isolated and reseeded for a second single-cell cloning step. The cells were kept in normoxia (3). c-Myc levels were analyzed at each stage by Western blot. The final single-cell clones were used for analysis of replicative profiles. (**B**) HA1EB Empty and c-Myc overexpressing cells analyzed for endogenous c-Myc mRNA levels (targeting 5′UTR). (**C**) MTH1 mRNA levels in HA1EB cells. (**D**) Cyclin E1 mRNA levels in Empty-3/4 and c-Myc-3/4 cells, (B-D) n=3. (**E**) Levels of the indicated proteins in HA1EB cells.

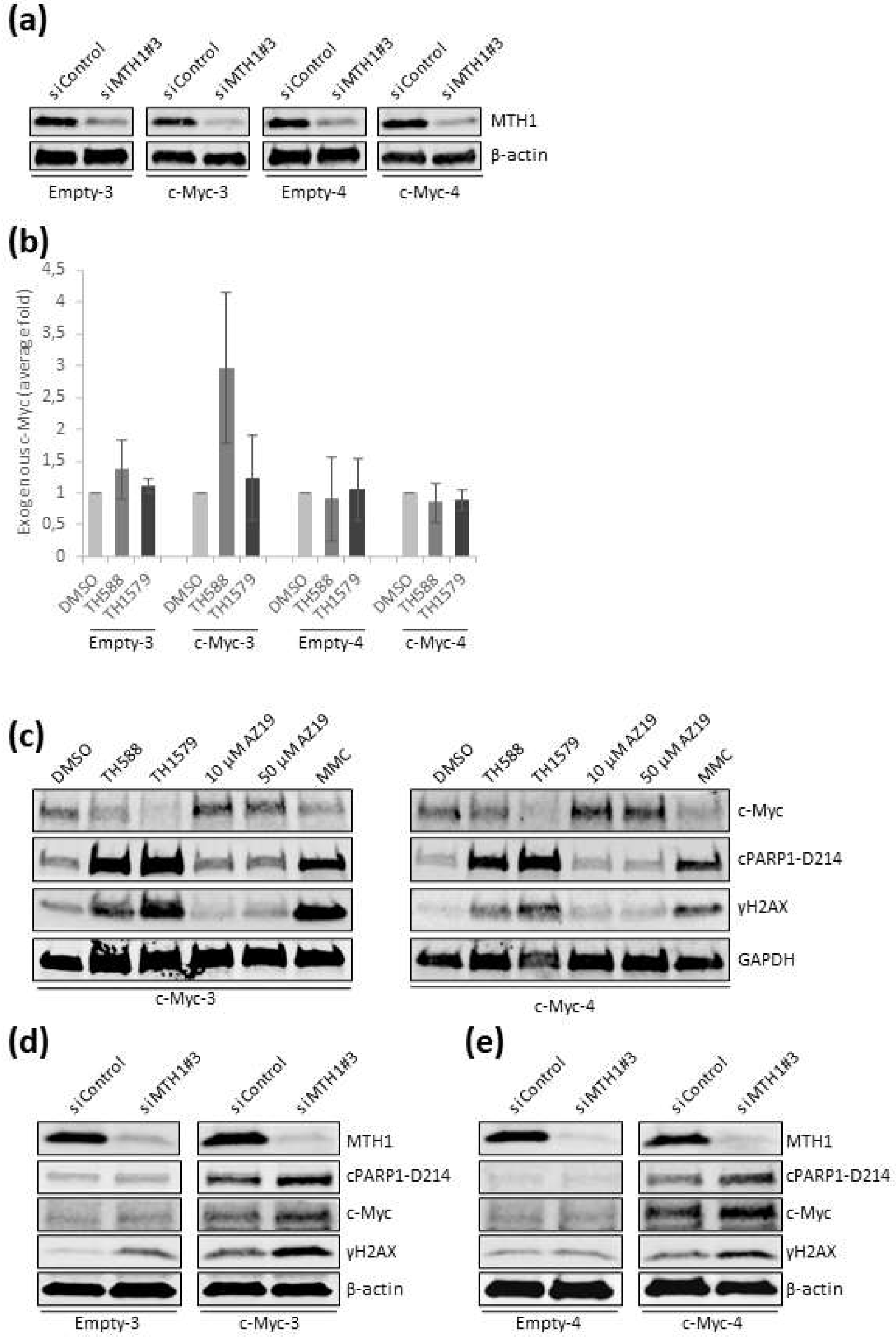

Figure S3. Non-toxic MTH1 inhibitors or MTH1 silencing do not decrease c-Myc levels. (**A**) HA1EB cells (Clones 3 and 4), were transfected with 10 nM siRNA for 72 hours followed by blotting for the indicated proteins. (**B**) Exogenous c-Myc mRNA levels were measured 24 hours post 5 μM TH588 or 0.5 μM TH1579 in HA1EB cells (Clones 3 and 4), n=2 ± S.E.M. (**C**) c-Myc-3 and -4 cells were treated with 5 μM TH588, 0.5 μM TH1579, 10 μM AZ19, 50 μM AZ19 or 4 μg/ml mitomycin C (MMC) for 24 hours. The indicated proteins were studied. c-Myc-3 (**D**) or c-Myc-4 (**E**) were transfected with 10 nM siRNA, harvested 72 hours later and the expression of indicated proteins was analyzed.

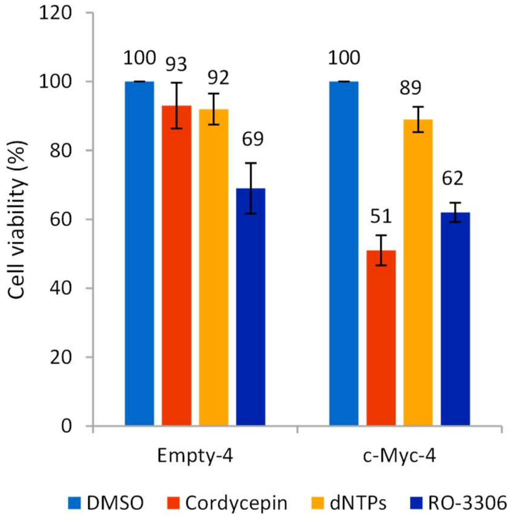

Figure S4. Viability of used inhibitors in Empty-4 and c-Myc-4 HA1EB cell lines. (A-B) Empty-4 and c-Myc-4 cells were seeded and following 24 hours, the cells were treated with DMSO ± co-addition of cordycepin (50 μM), dNTPs (50 μM), RO-3306 (5 μM). 10 μg/ml resazurin was added for 4 hours and viability was determined (values are normalized to DMSO control), n=2 ± S.E.M.

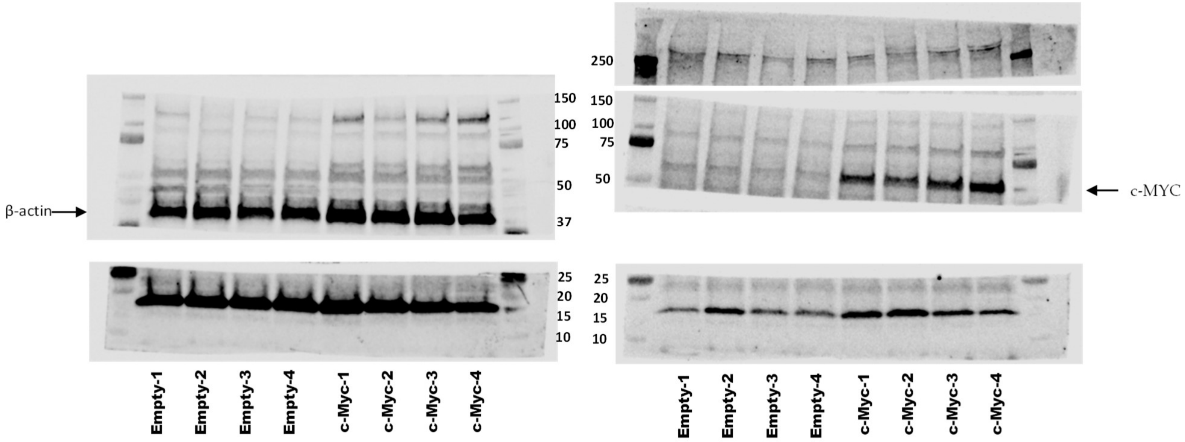

Figure S5. Uncropped Western blots for figure 1b.

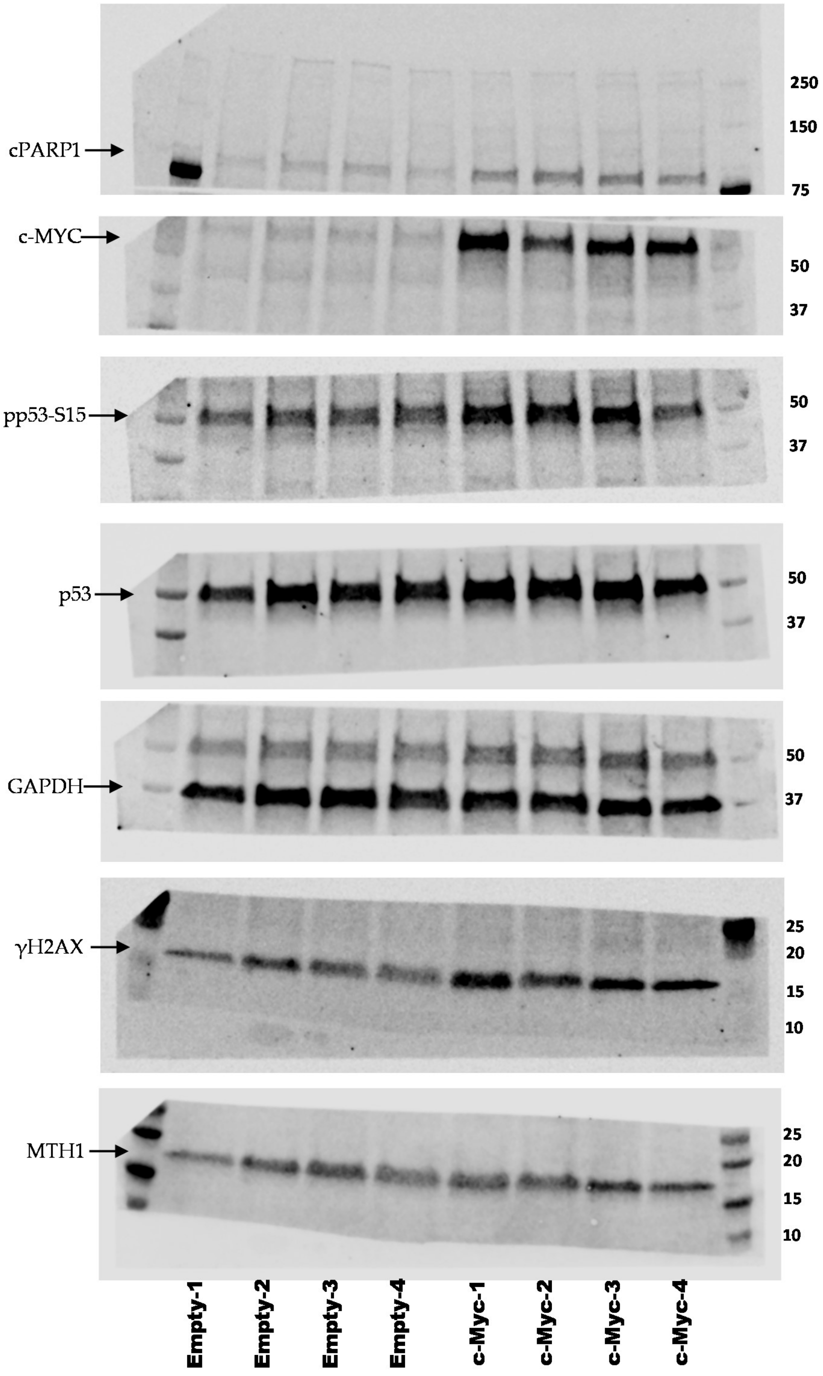

Figure S6. Uncropped Western blots for figure S2e.

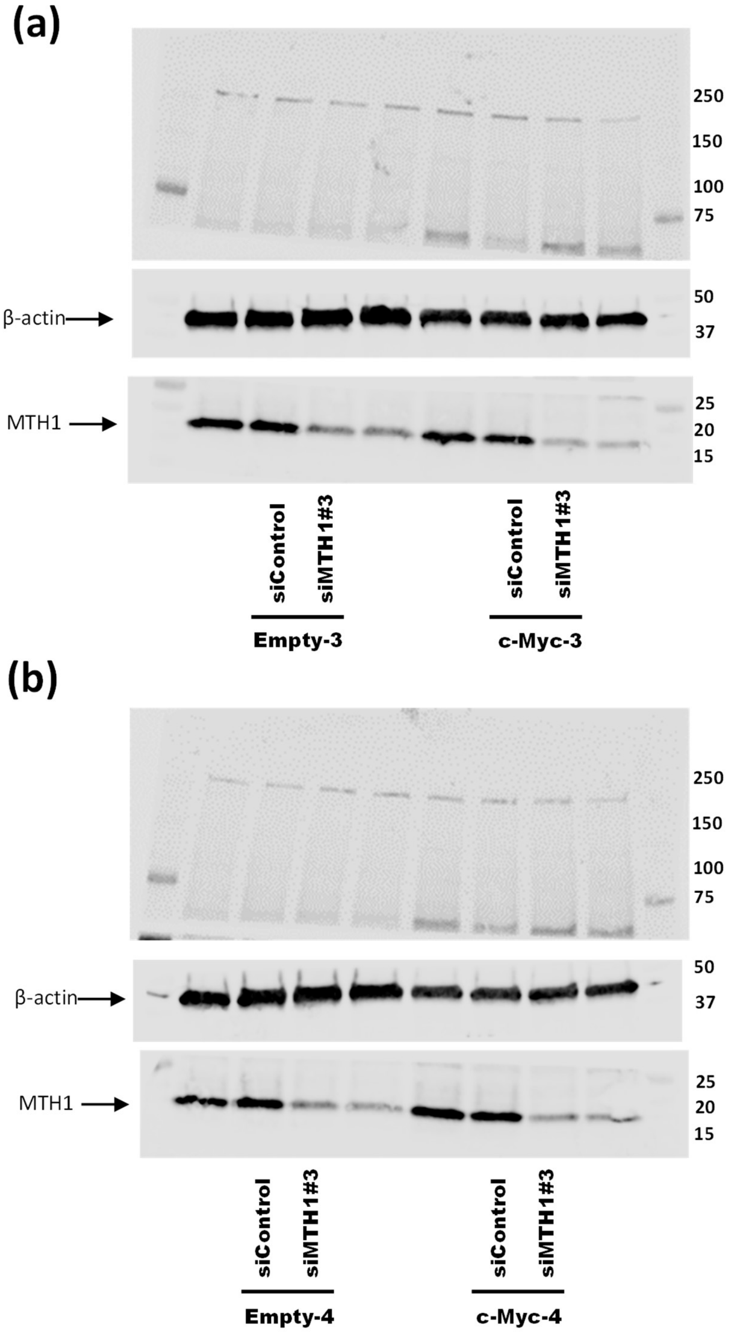

Figure S7. Uncropped Western blots for figure S3a.

(a)

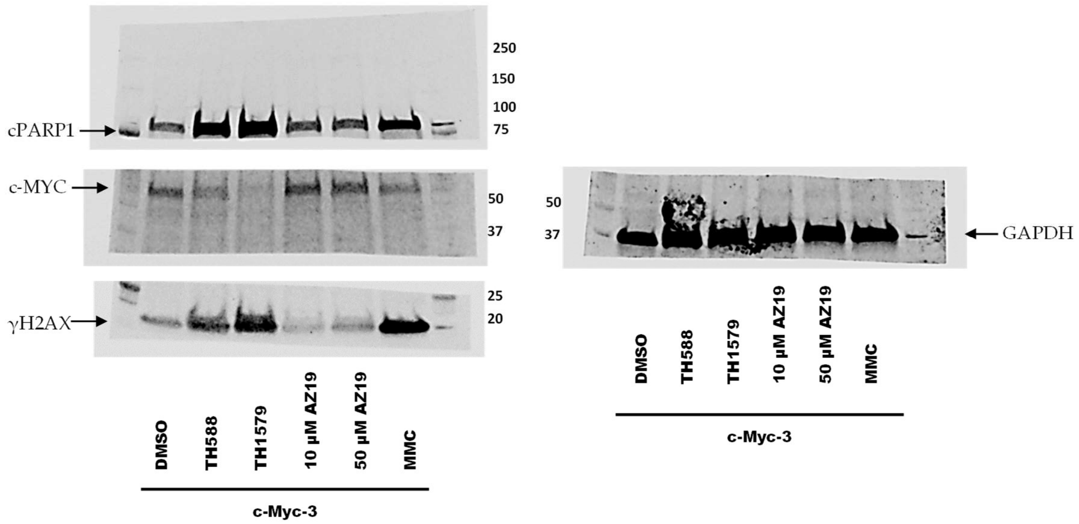

(b)

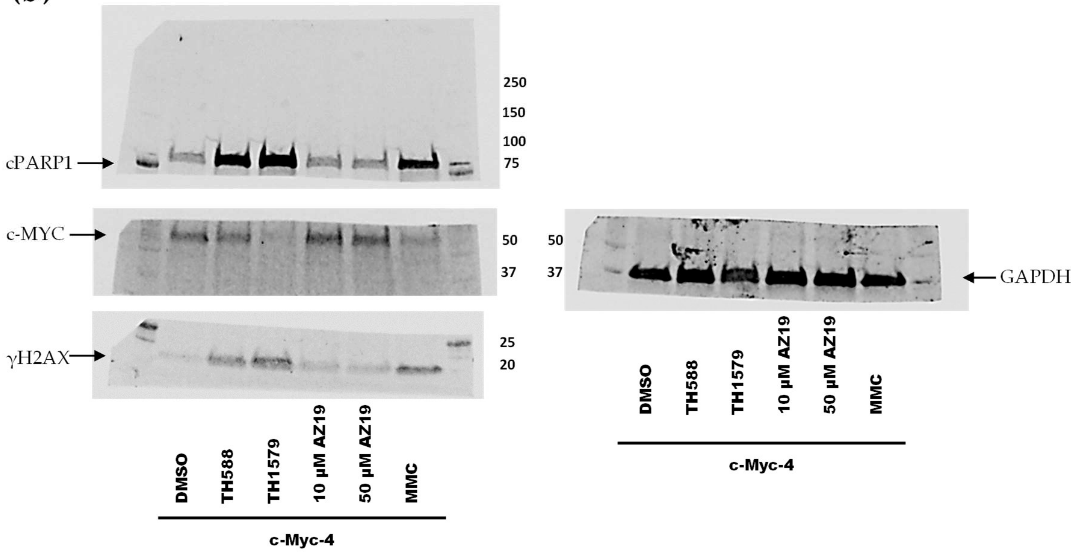

Figure S8. Uncropped Western blots for figure S3c.

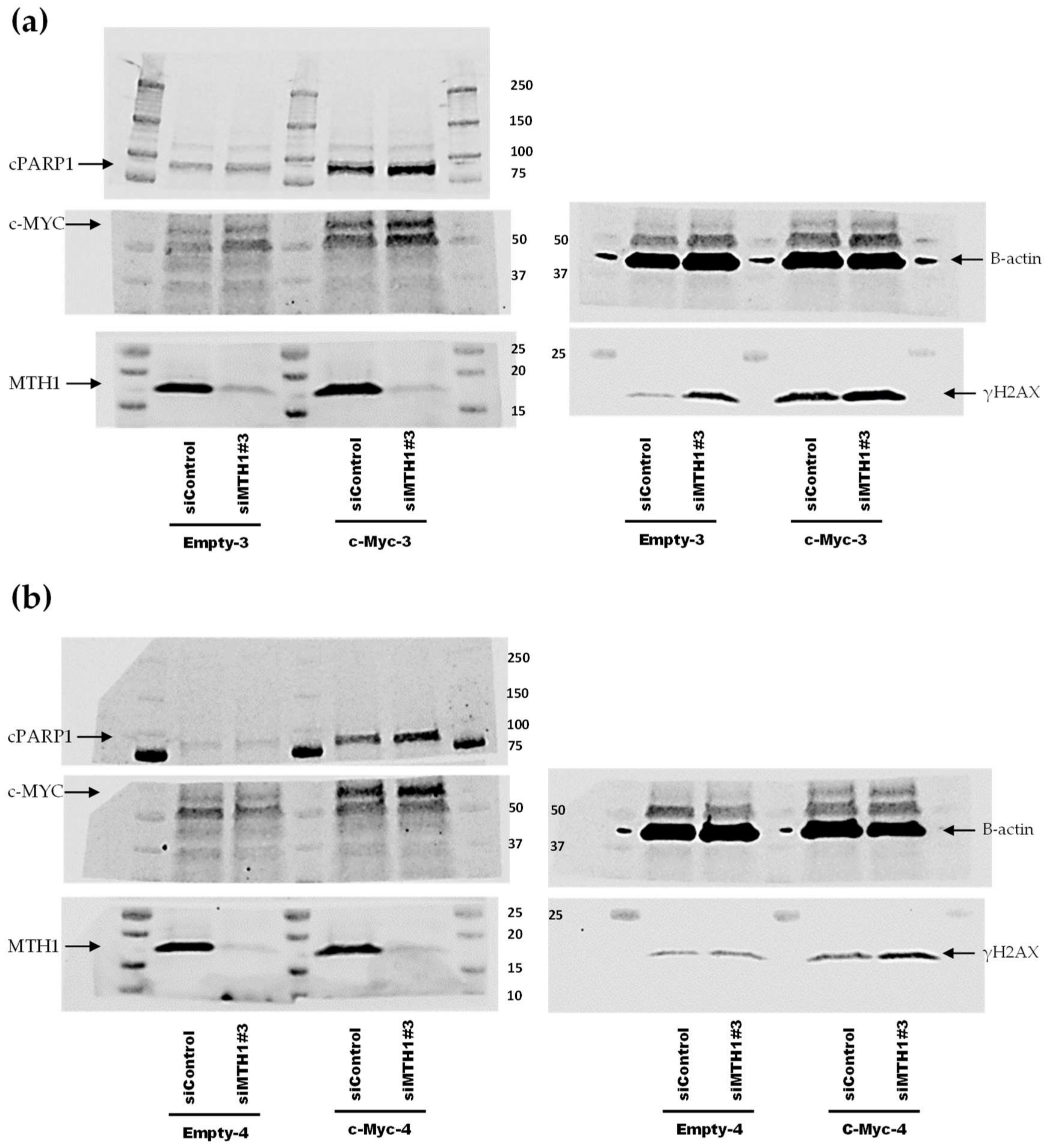

Figure S9. Representative uncropped Western blots for figure S3d-e.

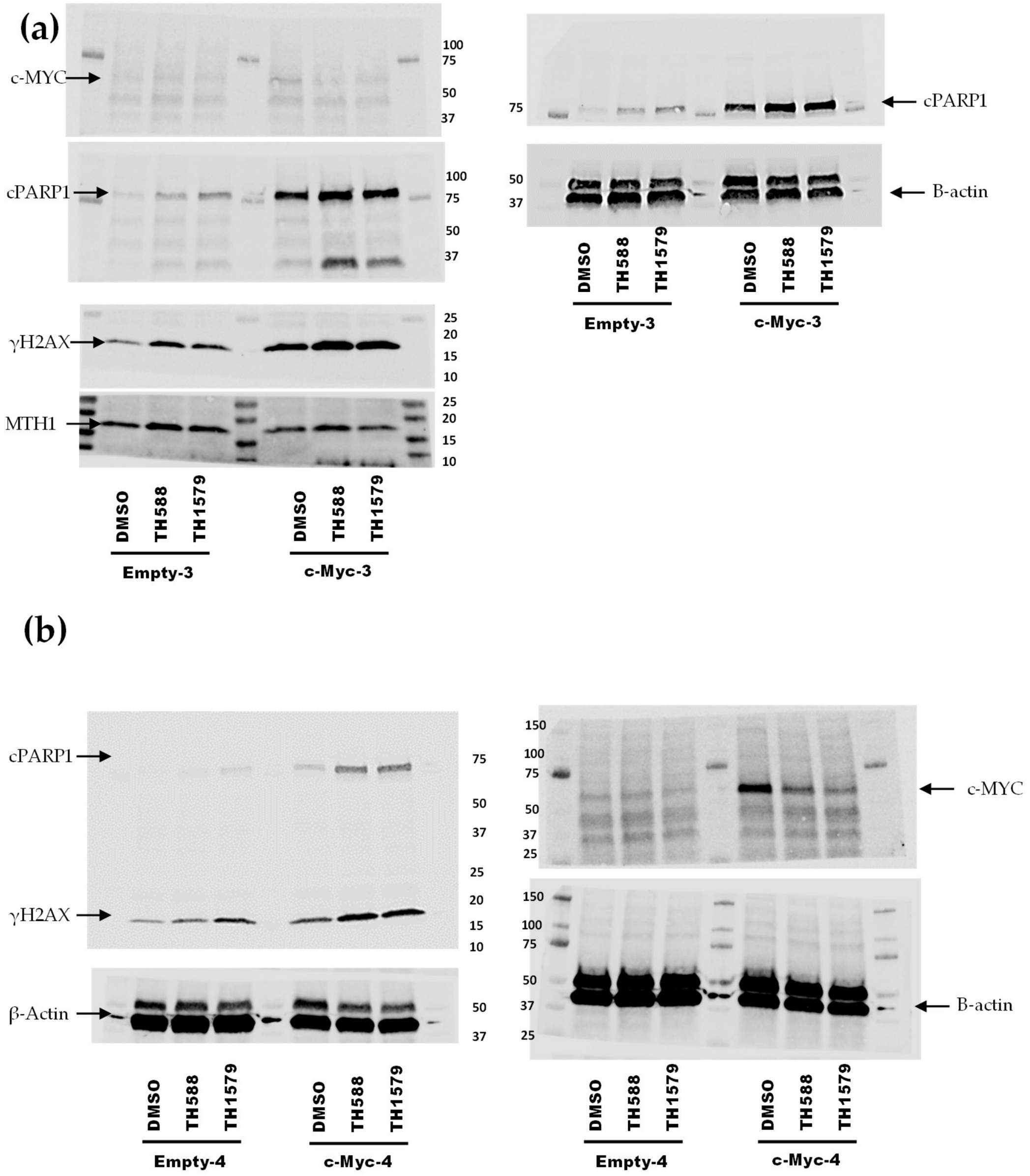

Figure S10. Representative uncropped Western blots for figure 5a.

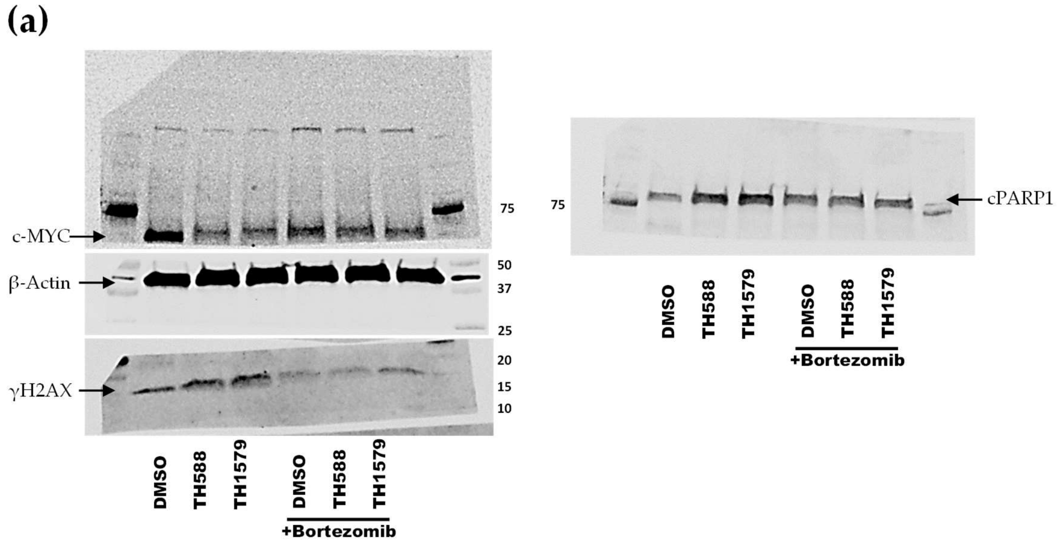

Figure S11. Uncropped Western blots for figure 5b.

(a)

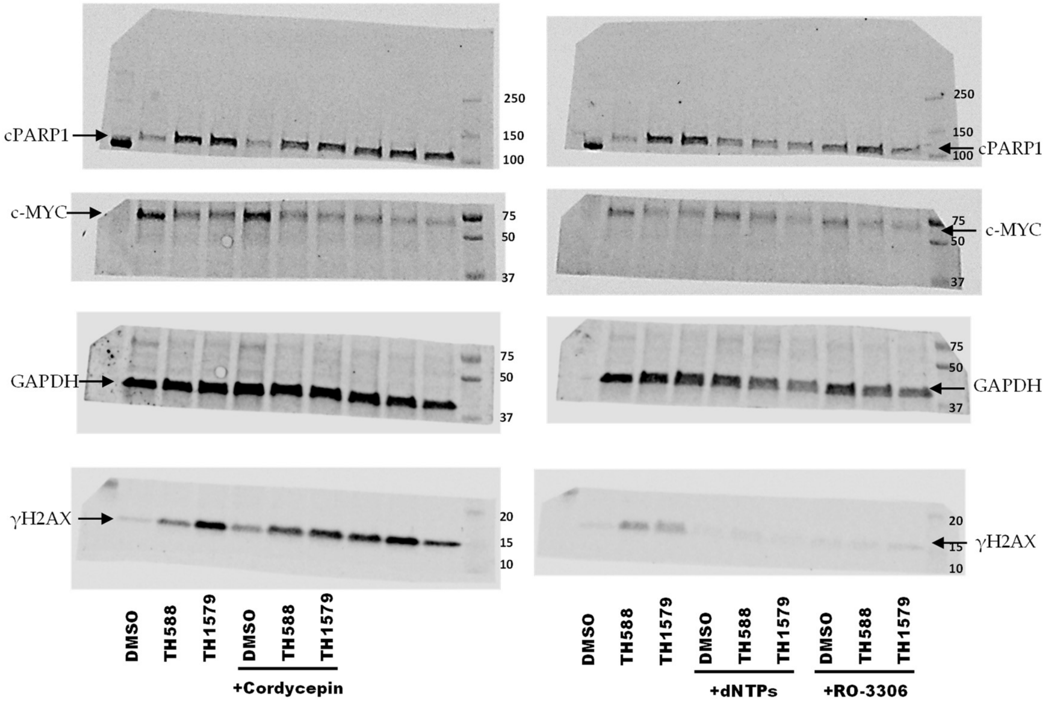

Figure S12. Uncropped Western blots for figure 6a.
